# Covid‐19 will Change the Agri‐food System – but how?

**DOI:** 10.1111/1746-692X.12276

**Published:** 2020-10-08

**Authors:** Krijn Poppe

**Affiliations:** ^1^ Wageningen Economic Research The Netherlands

## Abstract

The Covid‐19 pandemic has highlighted vulnerabilities in the agri‐food system and wider society. The elderly, in particular, have been overrepresented in intensive care units. The resulting economic crisis and accelerating geopolitical shifts will change the agri‐food system, but it is unclear how this will play out in detail. Two factors are important to consider: will societal priorities change and will the state become more interventionist? We examine these uncertainties via four scenarios from a Dutch perspective. ‘Business as usual’ is realistic if the crises are short and manageable. ‘Government Control’ is more state interventionist, after several decades of neo‐liberalism, with a greater focus on the economy and employment as the agri‐food system is confronted with a long recession. ‘Regional Communities’ is where there is a long period of echo‐pandemics, in which a flourishing community spirit, the attention to nature and a healthy living environment with healthy food are permanent and short supply chains and multifunctional agriculture gain ground. In ‘Green High‐Tech Transformation’, the most extreme scenario, the state and technical innovation take on a much larger role in society and our views on our lifestyle change. These trends reinforce each other and the government is tasked with creating a new economy. The scenarios are not predictions but can be used to structure thought and discussion on the way forward.

## Abstract (fr)

La pandémie de Covid‐19 a mis en évidence les vulnérabilités du système agroalimentaire et de la société en général. Les personnes âgées, en particulier, sont surreprésentées dans les unités de soins intensifs. La crise économique résultant de la pandémie et l'accélération des changements géopolitiques vont changer le système agroalimentaire, mais on ne sait pas comment exactement. Il est important de considérer deux questions : les priorités sociétales changeront‐elles et l’État deviendra‐t‐il plus interventionniste ? A l'aide de quatre scénarios, nous examinons ces incertitudes d'un point de vue néerlandais. Le « statu quo » est réaliste si les crises sont courtes et gérables. Le scénario « contrôle gouvernemental » envisage un État plus interventionniste après plusieurs décennies de néolibéralisme, avec une plus grande attention portée sur l’économie et l'emploi, le système agroalimentaire étant confronté à une longue récession. Le scénarios « communautés régionales » envisage une longue période d’écho‐pandémies dans laquelle un esprit communautaire florissant, l'attention à la nature et un environnement de vie sain avec des aliments sains se pérennisent, et les chaînes d'approvisionnement courtes et l'agriculture multifonctionnelle gagnent du terrain. Dans le scénario le plus extrême de « Transformation verte par les technologies de pointe verte », l’État et l'innovation technique jouent un rôle beaucoup plus important dans la société et nos opinions sur notre mode de vie changent. Ces tendances se renforcent mutuellement et le gouvernement est chargé de créer une nouvelle économie. Les scénarios ne sont pas des prévisions mais structurent la réflexion et la discussion sur la voie à suivre.

## Abstract (de)

Die Covid‐19‐Pandemie hat die Schwachstellen im Agrar‐ und Lebensmittelsystem und in der Gesellschaft insgesamt aufgezeigt. Vor allem ältere Menschen waren auf den Intensivstationen überrepräsentiert. Die aus der Pandemie resultierende Wirtschaftskrise und die sich beschleunigenden geopolitischen Verschiebungen werden das Agrar‐ und Lebensmittelsystem verändern. Dabei ist noch unklar, wie sich diese im Einzelnen auswirken werden. Zwei Faktoren sollten in dieser Hinsicht Berücksichtigung finden: Werden sich die gesellschaftlichen Prioritäten ändern und wird der Staat stärker eingreifen? Wir untersuchen diese Unsicherheitsfaktoren anhand von vier Szenarien aus niederländischer Sicht. „Business as usual” ist realistisch, wenn die Krisen kurz und überschaubar sind. Die „Regierungskontrolle” wird nach mehreren Jahrzehnten Neoliberalismus zunehmend staatlicher und interventionistischer werden. Hierbei werden Wirtschaft und Beschäftigung stärker im Fokus stehen, da das Agrar‐ und Lebensmittelsystem mit einer langen Rezession konfrontiert sein wird. „Regionale Gemeinschaften” werden dort entstehen, wo es einen lang andauernden Zeitraum mit Echo‐Pandemien gibt. In den „Regionalen Gemeinschaften” werden ein blühender Gemeinschaftssinn, Achtung der Natur und ein gesundes Lebensumfeld mit gesunden Nahrungsmitteln von Dauer sein. Dazu gewinnen kurze Lieferketten und eine multifunktionale Landwirtschaft an Boden. Bei der „Grünen High‐Tech‐Transformation”, dem extremsten Szenario, nehmen der Staat und technische Innovationen eine viel größere Rolle in der Gesellschaft ein – und ebenfalls in Bezug auf unsere Ansichten über die Änderung unseres Lebensstils. Diese Trends verstärken sich gegenseitig, wobei der Regierung die Aufgabe zukommt, eine neue Wirtschaftsform zu entwickeln. Die genannten Szenarien stellen keine Vorhersagen dar, sondern strukturieren Gedanken und Diskussionen beim weiteren Vorgehen.

The Covid‐19 pandemic will change the agri‐food system, but it is unclear how this will play out in detail. Two questions are important to consider: will societal priorities change, and will the state become more interventionist? To examine these issues, we present here four scenarios from a Dutch perspective.

## Health crisis

Covid‐19 is primarily a health crisis that has clear interfaces with the food system. It is a zoonosis that seems to have first arisen possibly from the illegal trade of exotic wildlife in Wuhan, China. However, it is being cited by critics of the Dutch livestock industry who highlight zoonosis risks in the Netherlands, such as a recent local outbreak of Q‐fever from goats that killed more than 75 people and disabled many. This year several mink farms have seen their animals killed in a stamping out programme due to transmission of Covid‐19 infections between animals and humans.

The Dutch region most affected by Covid‐19 and the zoonoses in goats and mink is Brabant, the heart of the pig industry. Problematic air quality, to which the livestock industry contributes with ammonia emissions and fine particles, is also an issue in Brabant. There are indications that air pollution plays a role through transmission of infection or because of relatively poor health conditions (*The Economist*, 2020; Wu *et al*., 2020).

A second notable interface between Covid‐19 and the food system is that many of the intensive care patients in hospitals are at greater risk due to obesity and/or lifestyle related issues. Whereas healthy young patients have a relatively high probability of not being hospitalised after catching Covid‐19 that is not the case for those who are older or in relatively poor health, which is often related to diet and lifestyle.

**Figure:**
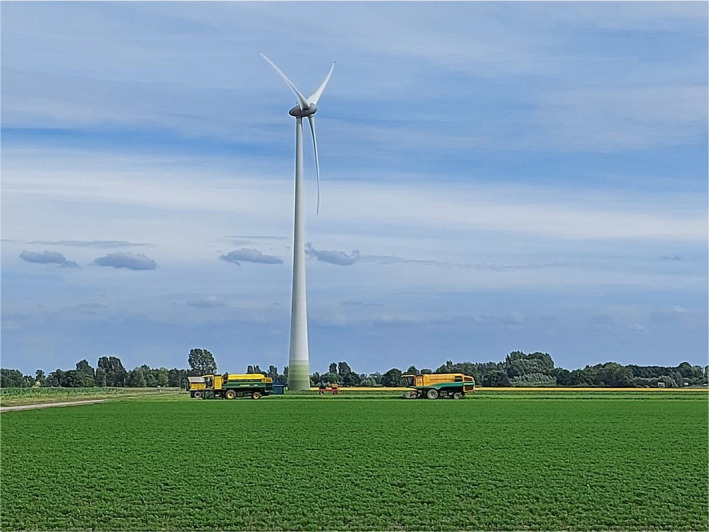
Social distancing is also needed in harvest operations.

These behavioural, lifestyle, health and environmental issues fit neatly into an ongoing analysis of the food system and the need for transition to healthy and sustainable food systems that researchers have been engaged in for some time. The negative effects occur at the weakest points, at the beginning and end of the chain but politicians find it difficult to address and correct these weaknesses (De Schutter, 2017; Rli, 2018).

## Economic and political crisis

The health crisis and the necessary lockdown measures quickly turned into a worldwide economic crisis of unprecedented magnitude. Severe recessions and sharply rising unemployment are already with us and are likely to persist. And if all that is not enough, there is also the risk that the world's geopolitical tensions will become sharper. Within the European Union, for example, cooperation was far from optimal as the pandemic took hold. The current financial measures will lead to much higher levels of debt; and this could in turn lead to a political and financial crisis in the EU. Different views among EU countries on the opportunities for a Green Deal on climate – and other environmental policies – can be expected.

“Pouvons‐nous utiliser ce type de cadre stratégique pour tracer l'avenir que nous voulons vraiment ?”

Internationally, Asian countries were better prepared after their Sars and Mers experiences. On the other hand, populist leaders apparently have had great difficulty (and less inclination) in intervening quickly and appropriately, with serious adverse consequences. The question is what all this means for the balance of power in the world, international trade and our thinking about the role of the state; and indeed, for the future of agri‐food systems.

## Market solutions and state actions

When it comes to our own (Dutch) agri‐food system it is still very difficult to identify the things that will be seen as ‘before Corona’ and those predicted as ‘after Corona’. In the short term, several different agri‐food chains were severely disrupted, for example:

- the markets for cut flowers and French fries (potatoes) collapsed;
- it has proved difficult to shift flows from the out‐of-home food market to supermarket distribution channels or to online; and
- experienced seasonal workers from Eastern Europe suddenly failed to show up and had to be replaced by unemployed catering staff.

**Figure:**
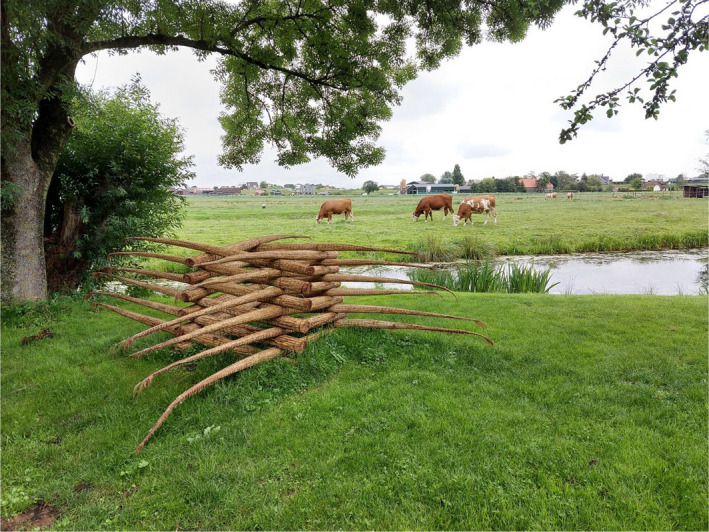
Local landscapes with art and cows gain interest in times of Covid‐19.

However, some effects are likely to be temporary, as markets react and aim to return to the pre‐Corona situation, but some will have more permanent impacts. Doing more digital business, from video conferencing to ordering food online, could stay with us now that we have been forced to learn how to do it. It is less clear if this also applies to the increased demand for more sustainable and healthier products and, for example, visits to the local farmer markets.

At the governance/institutional levels of the economy, changes can also be expected (Williamson, 2000). We can anticipate that companies will ask themselves some pertinent questions in this regard. CEOs will have to consider if just‐in‐time management paradigms should be extended to just‐in‐case risk‐mitigation (resilience) measures. Slaughterhouses in particular will need to take measures to avoid being confronted with further outbreaks of Covid‐19.

In the longer term there is considerable uncertainty about the likely role of the state. Its strengthened role in dealing with the pandemic and the economic crisis could lead to opportunities for state actions. In the past we have only experienced a light role for governments to help people with their health and lifestyle challenges. Now, however, the pandemic has led to growing awareness that, in addition to its role in the development, testing and approval of medicines, more state attention may well be needed in the area of preventive health care.

The same holds for climate and biodiversity concerns. Is a ‘green recovery’ the way forward? Are citizen preferences changing following the Covid‐19 experience? Speculation about the future is perhaps greatest in this area. Some think that we will adopt more community spirit, discovering more enjoyment from our own gardens, giving more attention to healthy and sustainable lifestyles. Others point to the fact that many young people, who have been striving to enhance the welfare of the older generation, will demand the same commitment from the community and governments to prevent what they see as the next big crisis namely, climate change. But just as easily, hedonistic behaviour could return to make up for lost time despite, or as a counterbalance to, unemployment. After all, the Spanish flu (1918–1920) was not an obstacle for the hedonism of the ‘Roaring Twenties’ – although of course this ended in a deep and prolonged depression.

My view, after six months of Covid‐19 experience, is that markets will adapt to the new economic, social and political environment. However, the biggest uncertainties for the agri‐food chain will be in the future role of the state – interventionist or neo‐liberal – and in the preferences of citizens concerning their living environment and greener lifestyles.

## Scenario analysis

**Figure 1:**
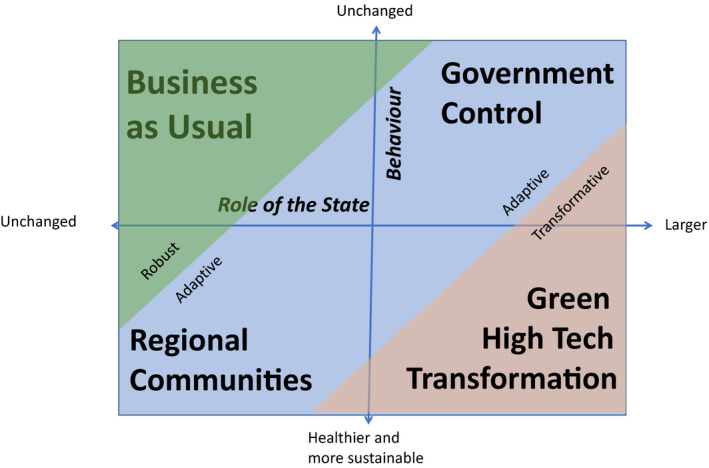
Potential post‐Covid‐19 outcomes for the Netherlands

The large uncertainties mentioned above are a good reason for undertaking scenario analysis: see for example, EU SCAR (2015) and Van der Heijden (2005). Figure 1 highlights four scenarios showing combinations of the ‘role of the state’ and ‘behaviour’, in a Dutch context.

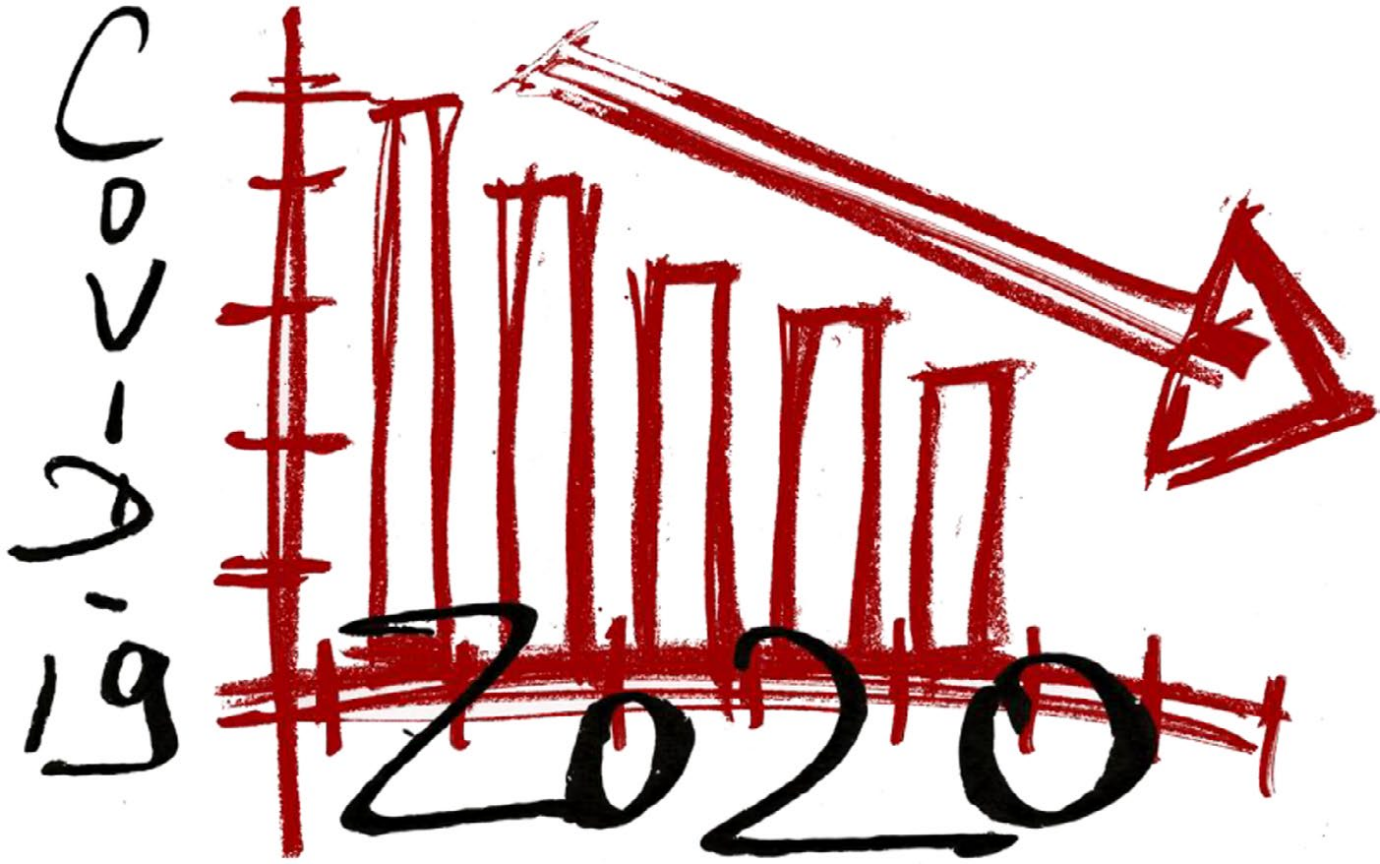

“Können wir diese Art von strategischem Referenzrahmen nutzen, um die Zukunft zu entwerfen, die wir wirklich wollen?”

- **Business as usual**: we foresee little change in our lifestyle and the role of the government in society. We quickly return to our old lifestyle of the ‘individualised society’. The government manages the recession, but it turns out to be mild with a ‘V‐shaped’ recovery and companies quickly repay loans. There is no incentive for government to be an activist shareholder. The unchanging public attitude means that there is little political pressure to make progress with climate and other environmental issues. This is not to say that nothing changes, for example: there will be more online sales along with risk management for pandemics (just-in‐case scenarios); production will be less concentrated with companies having plants in several countries, which apparently could be managed with video‐conferencing etc. This can lead to substantial changes, which potentially could make the agri‐food system more resilient in terms of robustness, adaptability and transformability (WUR, 2018). However, the Netherlands overall is not very different in this scenario.
- **Government Control** is a scenario in which our lifestyle changes little, but the government is given stronger power. The neoliberal consensus that Thatcher and Reagan embraced in the crises of the seventies moves into the background. We value stronger government again as an organiser of society, given its commitment to handling the pandemic and recession. The Covid‐19 virus remains for some time in our midst and the economic crisis is deep and long (‘L‐shaped’). The government will regulate the labour market to reduce income disparities and increase minimum wages. There will be more investment in health and education. To fund these changes taxes go up. Work, work, work is the adage and this is partly fulfilled via house construction, which would be mainly in ‘garden villages’ on agricultural land. The European Green Deal would be very much weakened so as not to disrupt the competitive position with other continents. However, part of the public works will have to do with climate and energy issues, given that international agreements apply and NGOs take government to court and find the judges on their side. Farmers who think that the environmental brake on production is released, given the attention to employment, will be disappointed. Environmental quotas will be transferred from agriculture to housing and industry. A circular agriculture economy is getting closer, for example through lower stocking density in livestock farming. There are more opportunities for horticulture as a healthier alternative within the agri‐food system. In terms of resilience thinking this scenario could be characterised as adaptive: not only do certain agri‐food food chains adapt to become more robust, but the government makes the overall agri‐food system itself more adaptive by fitting it within the available environmental space. These adaptations make the system less vulnerable to internal and external threats.
- **Regional Communities** is a scenario in which we actually adjust our lifestyle, but neo‐liberal politics continue to dominate. The flourishing community spirit, the attention to nature and a healthy living environment with healthy diets are permanent. The focus on inland or near‐by tourism is gaining in importance, first due to more waves of the virus and then partly due to the levels of unemployment and low‐income growth (‘W‐shaped’ recession). All this offers opportunities for short chain multifunctional and organic agriculture. Social coherence is considered important, which is reflected in, for example, regional products and authenticity. Labour costs remain low, as there is an inflow of workers from Member States having economic difficulties. There is regional political discussion on environmental aspects of agriculture, but the focus is on negative externalities like odours and emissions of ammonia and particulate matter, rather than on climate, soil or nitrogen that have less direct effects on neighbours. The large, export‐oriented agri‐businesses and the associated farmers therefore experience little opposition. The agri‐food system includes a regional component with multifunctional farmers and short supply chains, but there is also ‘conventional’ farming and multinationals with a strategy of scaling up to produce cheaper products for bargain hunters and for export. In terms of resilience thinking this scenario can also be characterised as adaptive: it is not only certain agri‐food chains that adapt to become more robust, but the overall agri‐food system itself is more diverse and adapts to new societal values.
- **Green High‐Tech Transformation** is a more extreme scenario. As in the national reconstruction of the 1950s, and the Government Control scenario above, the state takes on a much larger role in society. At the same time, our views on our lifestyle and how we want to interact with each other change. These two trends reinforce each other and the government is tasked with overseeing the creation of a radically different socio‐economic system and its governance. From the old economy we only save what we want to take into the future. To be prepared for the next crisis, the government is tasked with a strong climate policy. Given the success of the apps that intelligently managed our behaviour during the pandemic lockdown, ICT solutions are now fully deployed in society, partly at the expense of privacy. Farmers will be required to make public their use of pesticides and other environmental indicators. The great social experiment that was the Covid‐19 crisis taught government that consumer behaviour can indeed be influenced, or even controlled. These new tools are used to actively nudge a transition that replaces animal with vegetable protein. A meat tax is introduced and partially helps to cover government deficits. Some Covid‐19 tracking apps are expanded with a module to link food intake and health status so that your smartphone turns red, orange or green each week, and your doctor can read these signals with artificial intelligence software. To overcome privacy concerns, the government regulates and transforms the tech companies and platforms into a semi‐public utility. In the same way as the self‐employed face heavy competition and brokerage fees in the platform economy, farmers become the executive arm of multinational food processors; and it will be more effective for governments to force these companies to report on true costs than to subsidise and regulate farmers. As in the 1950s, independent and accurate cost‐based prices are calculated for efficient and effective government interventions to support/regulate farming operations. Robot technology is penetrating and replaces a large part of mainly immigrant seasonal work. This is particularly strong in horticulture because we will eat more healthily and want to keep this affordable for lower economic classes as operational labour is becoming more expensive. As a result of all innovations, the economic crisis dissipates and wages rise, further boosting the incentives towards automation and robotisation. The combination of changing lifestyles, increasing incomes and a steering/controlling government means that the size of the Dutch agricultural sector is adapted to the available and decreasing environmental space. At the same time, a lot of new technology is being developed that is being exported. The agri‐businesses increasingly see themselves as a (Northwest) European company and work in symbiosis with many smaller start‐ups that they sometimes collaborate with and perhaps buy to give scale. In terms of resilience, this scenario focuses on quite radical transformation.

**Figure:**
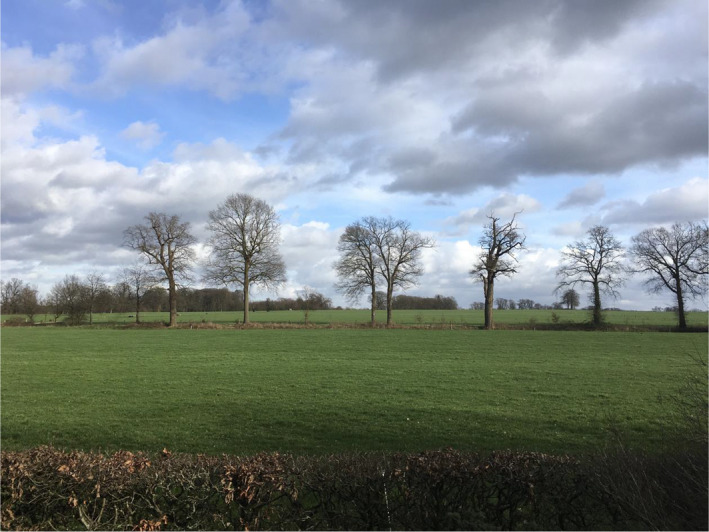
The future of the countryside depends on the post‐Covid‐19 outcome.

## Achieving consensus and charting a course

These scenarios, of course, are not predictions of what will happen in practice. That is not their intention. However, they are important in helping to structure thought and encourage discussion about the strategic directions or options for society; and in turn the implications for key sectors including of course the agri‐food system. For example, can we use this kind of strategic framework to map out the future we really want and what we mean when for example we talk about a green recovery? Policy makers and leaders in key sectors need at this juncture in our European – and indeed global – history to engage actively in this high‐level thinking and discussion and try to achieve a consensus as to the best way forward for a ‘green recovery’.

**Figure:**
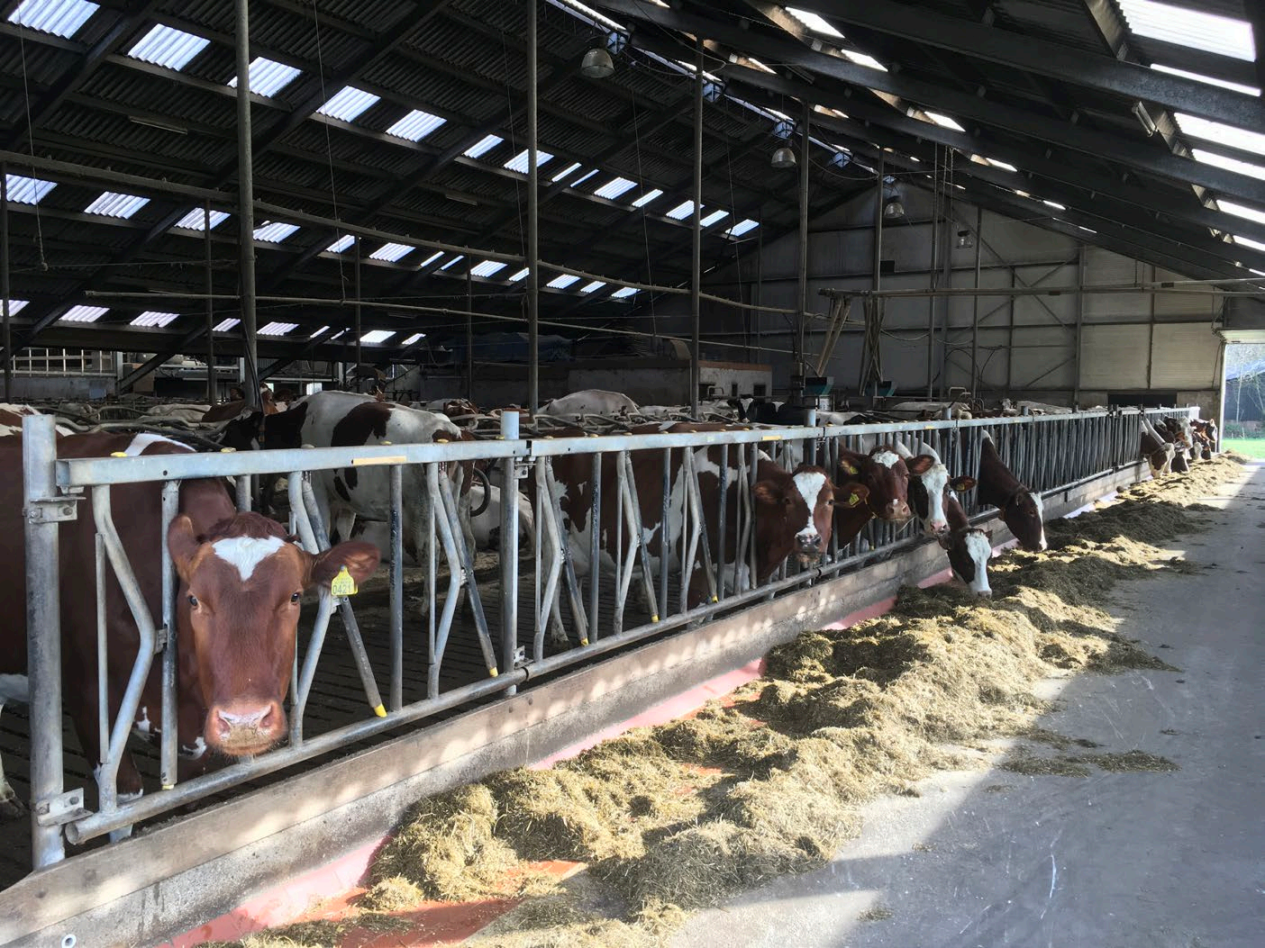
Will farmers become the executive arm of multinational companies?”.

“Can we use this kind of strategic framework to map out the future we really want?”

**Editor's Note:** The journal would welcome further contributions outlining your thoughts or scenarios on how you see the agri‐food system evolving following Covid‐19, either at your national level or more generally.
